# Equine Adipose-Derived Mesenchymal Stem Cells: Phenotype and Growth Characteristics, Gene Expression Profile and Differentiation Potentials

**DOI:** 10.22074/cellj.2015.491

**Published:** 2015-01-13

**Authors:** Faezeh Alipour, Abbas Parham, Hossein Kazemi Mehrjerdi, Hesam Dehghani

**Affiliations:** 1 Department of Clinical Sciences, Faculty of Veterinary Medicine, Ferdowsi University of Mashhad, Mashhad, Iran; 2 Division of Physiology, Department of Basic Sciences, Faculty of Veterinary Medicine, Ferdowsi University of Mashhad, Mashhad, Iran; 3 Embryonic and Stem Cell Biology and Biotechnology Research Group, Institute of Biotechnology, Ferdowsi University of Mashhad, Mashhad, Iran

**Keywords:** Mesenchymal Stem Cells, Equine, Adipose, Characterization, Differentiation

## Abstract

**Objective:**

Because of the therapeutic application of stem cells (SCs), isolation and characterization of different types of SCs, especially mesenchymal stem cells (MSCs), have gained considerable attention in recent studies. Adipose tissue is an abundant and accessible source of MSCs which can be used for tissue engineering and in particular for treatment of musculoskeletal disorders. This study was aimed to isolate and culture equine adipose-derived MSCs (AT-MSCs) from little amounts of fat tissue samples and determine some of their biological characteristics.

**Materials and Methods:**

In this descriptive study, only 3-5 grams of fat tissue were collected from three crossbred mares. Immediately, cells were isolated by mechanical means and enzymatic digestion and were cultured in optimized conditions until passage 3 (P3). The cells at P3 were evaluated for proliferative capacities, expression of specific markers, and osteogenic, chondrogenic and adipogenic differentiation potentials.

**Results:**

Results showed that the isolated cells were plastic adherent with a fibroblast-like phenotype. AT-MSCs exhibited expression of mesenchymal cluster of differentiation (CD) markers (CD29, CD44 and CD90) and not major histocompatibility complex II (MHC-II) and CD34 (hematopoietic marker). Cellular differentiation assays demonstrated the chondrogenic, adipogenic and osteogenic potential of the isolated cells.

**Conclusion:**

Taken together, our findings reveal that equine MSCs can be obtained easily from little amounts of fat tissue which can be used in the future for regenerative purposes in veterinary medicine.

## Introduction

Cell therapy is considered a valuable strategy for the treatment of some untreatable diseases in veterinary medicine, especially in treating equine orthopedic diseases with particular attention to ligament and tendon injuries (1-4). It is widely believed that mesenchymal stem cells (MSCs) mediate tissue and organ repair by virtue of their multi-lineage differentiation potential and production of different factors including growth factors, cytokines and antioxidants (5). To date, many studies have reported the application of MSCs for the treatment of tendonitis (2, 3) and ligament disorders (6).

Equine MSCs have been isolated from a number of different tissues including bone marrow, fat tissue, peripheral blood, and extra-fetal tissue (2-5). Reduced immunogenic properties and immunosuppressive potentials of MSCs make them attractive for allogenic stem cell therapy (7). In spite of many similarities among MSCs derived from various sources, they have some differences. Bone marrow-derived MSCs have less proliferation and high osteogenic potential compared with umbilical cord- and adipose tissue-derived MSCs (AT-MSCs) (8). In fact, adult adipose tissue of various species is a suitable source of fibroblast-like precursor cells that have multipotential differentiation capacity (9, 10). Compared with bone marrow, more MSCs could be recovered from adipose tissue and because of this favorable characteristic, there has been a growing interest in the application of AT-MSCs for cell therapies compared to other sources (11-13).

Based on the current knowledge in the field of MSC research, the minimal criteria for confirmation of MSCs are 1) adherence to plastic when maintained under standard culture conditions, 2) differentiation potential towards osteogenic, chondrogenic and adipogenic lineages, and 3) expression of cluster of differentiation 29 (CD29), CD44 and CD90 and lack of expression of CD34, CD79 and major histocompatibility complex II (MHCII) (14, 15). However, the defining characteristics of MSCs are inconsistent among investigators and various methods developed to isolate and expand MSCs occasionally have significant differences among them (15). So, it seems that more investigation is needed to find reliable methods for isolation of equine MSCs.

In most previous studies, a 20 cm incision is usually made on skin to take 30-50 g fat for MSC isolation. Healing of this long incision needs much time and may increase infection incidence. In addition, the amount of available subcutaneous fat tissue is little in some cases especially in athletic horses. Thus, the aim of this study was to isolate enough number of MSCs from minimal sample sizes of fat and to determine some of their biological characteristics in comparison with other studies.

## Materials and Methods

All reagents were purchased from Sigma (Germany), unless otherwise stated.

### Tissue sampling

In this descriptive study, adipose tissue was collected from 3 crossbred mares aged between 3 and 10 years. The region above the dorsal gluteal muscle, at the base of the tail, was chosen as the adipose tissue collection site because of the availability of material, the absence of large veins and ease of access. Horses were restrained and sedated with Xylazin (0.5 mg/kg body weight intravenously) which was followed by shaving an area of approximately 20×20 cm on the paraxial caudodorsal gluteal region. After aseptically prepared, local anesthesia in a line with lidocaine 2% was performed and pain assessment was made by observing animal response to painful stimuli. An incision of approximately 4-5 cm in length was made parallel to and approximately 15 cm lateral to the spinal column, permitting visualization of a layer of adipose tissue between the skin and musculature. After dissection of the subcutaneous tissue, approximately 3-5 g of adipose tissue was collected and stored in a sterile 50 mL tube containing phosphate buffer saline (PBS) supplemented with penicillinstreptomycin (1%) and amphotericin (0.1%). The skin was sutured with nylon 2/0 in simple isolated stitches. Samples were immediately transported to the cell culture laboratory and processed within 12 hours. All procedures were approved by The local Ethics Committee of Ferdowsi University of Mashhad.

### Tissue sampling

In this descriptive study, adipose tissue was collected from 3 crossbred mares aged between 3 and 10 years. The region above the dorsal gluteal muscle, at the base of the tail, was chosen as the adipose tissue collection site because of the availability of material, the absence of large veins and ease of access. Horses were restrained and sedated with Xylazin (0.5 mg/kg body weight intravenously) which was followed by shaving an area of approximately 20×20 cm on the paraxial caudodorsal gluteal region. After aseptically prepared, local anesthesia in a line with lidocaine 2% was performed and pain assessment was made by observing animal response to painful stimuli. An incision of approximately 4-5 cm in length was made parallel to and approximately 15 cm lateral to the spinal column, permitting visualization of a layer of adipose tissue between the skin and musculature. After dissection of the subcutaneous tissue, approximately 3-5 g of adipose tissue was collected and stored in a sterile 50 mL tube containing phosphate buffer saline (PBS) supplemented with penicillinstreptomycin (1%) and amphotericin (0.1%). The skin was sutured with nylon 2/0 in simple isolated stitches. Samples were immediately transported to the cell culture laboratory and processed within 12 hours. All procedures were approved by The local Ethics Committee of Ferdowsi University of Mashhad.

### Isolation and expansion of putative MSCs

The collected fat tissues were subjected to successive washes with PBS containing antibiotic and fungicide in sterile falcon tubes. To isolate the cells, samples were frittered into small pieces using a No.15 scalpel blade and then were subjected to digestive action of 0.1% of type I collagenase supplemented with 1% bovine serum albumin (BSA) in an incubator at 37˚C for 2 hours. The content of the Falcon tube was then passed through a 70 μm cell strainer and the enzyme was neutralized with Dulbecco’s Modified Eagle’s Medium (DMEM) containing 10% fetal bovine serum (FBS) (Invitrogen, Germany). This solution was centrifuged with a relative centrifugal force of 600 xg for 5 minutes and the cell-containing pellet was resuspended in DMEM. Finally, the cell number was quantified in an improved Neubauer counting chamber and cell viability was assessed by the standard exclusion test using 0.4% trypan blue.

The cells were seeded in 25 cm^2^ flasks with a density of 8×10^4^ cells per cm^2^ and incubated in basic growth medium containing DMEM (high glucose) supplemented with 10% FBS, 1% penicillin- streptomycin and 0.1% amphotericin at 37˚C in 5% CO_2_. This initial phase of the primary cell culture was identified as passage 0 (P0). After 72- 96 hours, non-adherent cells were discarded and the medium changed twice a week. The cells were maintained in culture until they achieved 80% confluency and were examined daily using an inverted microscope. At day 21, the cells were harvested using 0.4% TrypLE enzyme (Invitrogen, Germany), reseeded at a density of 5000 cells/cm^2^ (P1) in new flasks for extensive cultivation and were then maintained in basic growth medium until P3.

### Assessment of cell proliferation

#### Growth curve and cell population doubling

To demonstrate the proliferation capacity of AT-MSCs, the cells of each horse at P3 were seeded in 12-well plates with a density of 30,000 cells per well. After 24 hours, the cells of 3 wells were daily trypsinized, counted and averaged from day 1 to 8 to determine the status of cell growth. Moreover, the population doubling time (PDT) of P3 cells was obtained according to the formula (T-T0) lg2/(lgNt–lgN0) where T0 is starting time of cell culture and T is ending time of cell culture, and N0 and Nt represent the cell number at the beginning and the end of culture respectively.

#### Colony forming units assay (CFU assay)

The cells at passage 3 from each horse were also seeded in 6-well plates with a density of 100 and 500 cells per well in three replicas. Fresh basic growth medium was added to each well and incubated for 12 days at 5% CO_2_ and 37˚C. Finally, the culture medium was aspirated, each well was washed with PBS and topped up with 1ml of 0.5% crystal violet solution and then incubated for 10 minutes at room temperature. After, each well was washed with a gentle stream of water under a running tap water. The number of 'darkly' stained colonies (greater than or equal to 20 nucleated cells) were counted under the phase-contrast microscope at low magnification and data are reported as colony-forming or plating efficiency (PE%) calculated as the percentage of the ratio of number of colonies counted to number of cells initially seeded.

#### Tri-lineage differentiation assay

In order to confirm that cultured cells, at the end of the P3, of the 3 horses belonged to the lineage of mesenchymal stem cells, a portion of the cell lineage was induced to osteogenic, adipogenic and chondrogenic differentiation as previously described (12, 16). Adipogenic and osteogenic differentiation was performed in a monolayer, whereas the chondrogenic differentiation took place in a pellet culture. Non-induced MSCs, cultured with basic growth medium only, were used as negative controls for each type of differentiation.

#### Osteogenic differentiation

Cells were initially seeded with a density of 300,000 cells per well in six-well plates. The cell monolayer, after reaching 50% of confluency, was cultured for 21 days in osteogenic medium containing DMEM (high glucose) supplemented with 10% FBS, 0.1 μM dexamethasone, 10 mM β-glycerophosphate disodium, 50 μM 2-phospho- L-ascorcbic acid trisodium salt, 0.1% amphotericin B and 1% penicillin/streptomycin.

At day 21, Alizarin Red staining was carried out to detect calcified extracellular matrix deposits. Following fixation with 10% neutral buffer formalin for 20 minutes, cell layers were washed 2-3 times with distilled water and 1.5 ml fresh 2% Alizarin Red solution (pH=4.1-4.3) was added to each well. Following incubation at room temperature for 20 minutes, the stain was removed and washed 3-4 times with water until the rinsed solution was clear. Alizarin Red forms complexes with calcium ions and mineral deposits, and makes a bright red color.

#### Adipogenic differentiation

Cells were initially seeded with a density of 300,000 cells per well in six-well plates. A monolayer of cells, after reaching 80% of confluence, was cultured for 21 days in adipogenic medium containing DMEM (high glucose) supplemented with 10% FBS, 1 μM dexamethasone, 10 μg/mL Insulin-Transferrin-Selenium-X (ITS) (Invitrogen, Germany), 0.5 mM 3-Isobutyl-1-methylxathine, 0.1 mM Indomethacin, 0.1% amphotericin B and 1% penicillin/streptomycin. Oil Red O staining was used to determine the intracellular lipid droplets and evaluate adipogenic differentiation. Briefly, the medium was removed and the cells were fixed using 10% neutral buffer formalin for 20 minutes at room temperature. The cells were then washed with distilled water and incubated with a 0.4% Oil Red O solution for 20 minutes.

#### Chondrogenic differentiation

Chondrogenic differentiation of P3 MSCs was performed in pellet culture. Chondrogenic differentiation medium consisted of DMEM (high glucose) supplemented with 10% FBS, 10 ng/mL human transforming growth factor β3 (TGF-β3), 10 ng/mL human bone morphogenetic protein 6 (BMP-6), 1% ITS, 0.1 μM dexamethasone, 50 μM 2-phospho-L-ascorcbic acid trisodium salt, 1 mg BSA, 0.1% amphotericin B and 1% penicillin/streptomycin. After incubation for 21 days, paraffin sections were prepared and stained with Alician blue.

### Gene expression analysis

#### RNA isolation and reverse transcription-polymerase chain reaction (RT-PCR)

AT-MSCs were examined for expression of surface markers using RT-PCR. Total RNA of MSCs at P3 of the 3 horses were extracted according to manufacturer’s instructions using the Tripure reagent (Roche, Germany) and quantified by a Nanodrop apparatus. After DNase treatment (Roche, Germany), one μg of total RNA was reverse-transcribed into cDNA with 0.5 μg oligo thymidine, 1 mM dNTP mix and 4 μl 5X reaction buffer in a final volume of 20 μl for 60 minutes at 42˚C followed by heating at 70˚C for 10 minutes to stop the reaction.

Specific primers for Glyceraldehyde 3-phosphate dehydrogenase (GAPDH), CD29, CD34, CD44, MHC-II and CD90 were designed based on the known sequences (Table 1). One μl of each RT reaction was used as template for PCR reactions (35 cycles) in a final volume of 25 μl with 0.2 mM dNTP mix, 20 pmol of each primer, 1.25 units Smart-Taq DNA Polymerase, 1.5 mM MgCl_2_ and 5 μl 10X PCR buffer. The amplification of the housekeeping gene *GAPDH* was used as a positive internal control for all samples to verify that the RT-PCR reactions were successful. Negative control reactions were performed similarly without the addition of a template and with the addition of RNA templates. Amplified cDNA were visualized on a 1.5% agarose gel stained with ethidium bromide. The molecular sizes of the transcripts were determined by comparison with size marker run together with the cDNA product.

**Table 1:** Nucleotide sequences of the primer sets used for RT-PCR


Genes	Gen bank accession number	Primer pairs	Annealing temperature	Amplicon size(bp)

**Equine GAPDH**	NM_001163856	F:TGTCATCAACGGAAAGGC	57	183
		R:GCATCAGCAGAAGGAGCA		
**Equine CD29**	XM_001492665	F:AATCGGGACAAGTTACCTCA	56	234
		R:CTTCCAAATCAGCAGCAA T		
**Equine CD34**	XM_001491596	F:TGATGAATCGCCGTAGT	56	204
		R:CGGGTTGTCTCGCTGA		
**Equine CD44**	NM_001085435	F:AACCTCGGGTCCCATAC	56	193
		R:TCCATTGAGCCCACTTGC		
**Equine CD90**	XM_001503225	F:AGAATACCACCGCCACA	57	155
		R:GGATAAGTAGAGGACCTTGATG		
**Equine MHC-II**	NM_001142816	F:GGAACGGGCAGCAGGACAT	57	184
		R:AAGCCATTCACAGAGCAGACCA		


GAPDH; Glyceraldehyde 3-phosphate dehydrogenase, CD; Cluster of differentiation, MHC-II; Major histocompatibility com-plex II and RT-PCR; Reverse transcription- polymerase chain reaction.

## Results

### Sample collection, cell isolation and expansion

**Fig 1:**
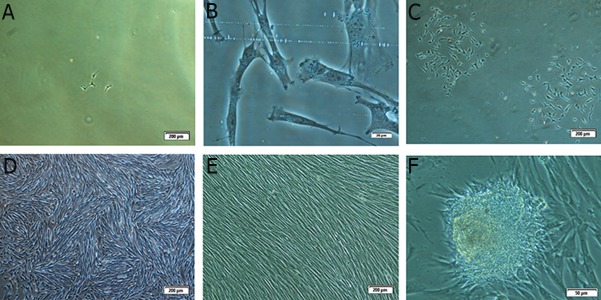
Morphological characteristics of putative mesenchymal stem cells in different days: A. Primary single adherent cells on the 4^th^ day after seeding (×10), B. Spindle and satellite cell morphology (×40), C. Cell colonies on the 7th day after seeding (×4), D. 80% cofluency at the end of primary culture on day 19 (×4), E. Uniform population of fibroblast-like mesenchymal stem cells (MSCs) at the end of second passage (×4) and F. Formation of some nodular cell aggregations in the primary culture (×20).

Collection of adipose tissue samples did not have any adverse effect on the donors. The amount of processed fat samples and total mononuclear cell yield in each sample are presented in table 2. In the first days of primary culture, single cells with satellite or spindle shape morphology were observed (Fig 1A, B). Dead cells floating in the medium were removed by changing the culture media on days 3 to 5 after seeding. Adherent cells proliferated and developed round colonies with fibroblast-like cells visible on several areas of culture surface one week after culture initiation (Fig 1C). About 18 to 21 days after initial cell seeding, they reached 70 to 80 percent confluency (Fig 1D) and the culture consisted of an almost homogenous monolayer of fibroblast-like cells (Fig 1E) with a number of nodule-like cell aggregates (Fig 1F). Finally, the cells were passaged and after 3 successive passages, a sufficiently near homogenous cell population was obtained to be used in the next phase of the experiment. Throughout the cultivation period, the cells maintained their fibroblastic morphology.

**Table 2:** Cell yield and growth characteristics of isolated MSCs from the 3 horses


	Age (Y)	Amount of processedfat sample (g)	Mononuclear cellyield/g fat tissue	Plating efficiency(CFU%, mean ± SD)	Doubling timeat P3 (h)

**Horse 1**	3	5	1.2×10^6^	5.33 ± 2.88	40.39 ± 1.75
**Horse 2**	6	3	6.66×10^5^	5.73 ± 2.53	46.75 ± 2.21
**Horse 3**	9	4	3.75×10^6^	-*	44.32 ± 3.22


*; Cultured cells were lost, MSCs; Mesenchymal stem cells, CFU; Colony forming unit, SD; Standard deviation and P3; Passage 3.

### Cell proliferation assay (growth curve, cell population doubling and CFU)

**Fig 2:**
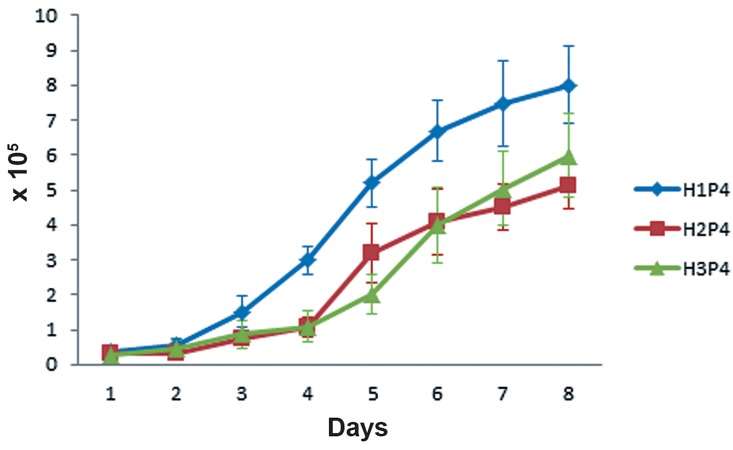
The growth curve of the P3 AT-MSCs belonging to the 3 horses. Cells rapidly enter the log phase after a brief lag phase and not reaching the plateau until day 8. P3; Passage 3 and AT-MSCs; Adipose tissue-derived MSCs.

**Fig 3:**
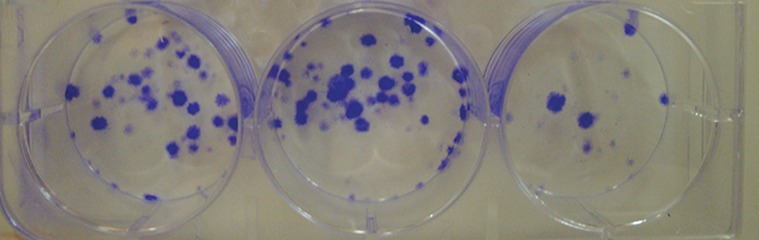
P3 cells of horse 1 are cultured (500 cells per well) in 3 replicates. Colony-forming units are visible (blue) using crystal violet staining after 12 days of culture. P3; Passage 3.

Growth curve of P3 cells was determined by counting viable cells for 8 continuous days (Fig 2). AT-MSCs showed high growth capacity in culture. They rapidly became adaptive to the culture conditions (during the first 3 days) and then started to enter the logarithmic phase (days 3-6). Even after day 7, they did not reach the plateau phase and their growth was continued with a decreased rate. PDT for P3 cells of the 3 samples were calculated. When the clonogenic capacity of the ATMSCs was analyzed using the CFU-F assay, results demonstrated the presence of clonogenic cell populations in the examined populations (Table 2). AT-MSCs formed CFU-F by 12 days after seeding (Fig 3). In wells with 100 cell density, almost no colony was formed. Unfortunately, cultured cells of horse 3 were lost.

### Isolated MSCs showed tri-lineage differentiation

**Fig 4:**
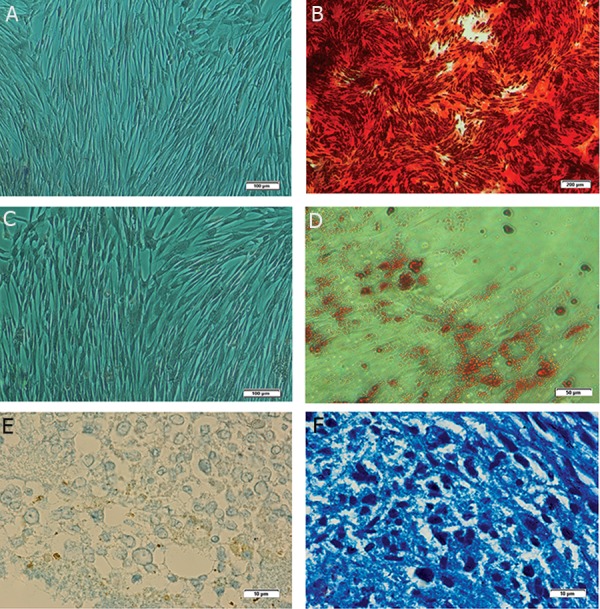
Representative tri-lineage differentiation of equine AT-MSCs. Alizarin Red staining of control group (A) (×10) and osteogenic treatment group (B) (×4). Oil Red O staining of control group (C) (×10) and adipogenic treatment group (D) (×20) where lipid droplets inside the cytoplasm are stained with Oil Red O dye. Alician blue staining of a pellet section of control group (E) (×100) and chondrogenic differentiation group (F) (×100) where proteoglycans are stained in the extracellular matrix. ATMSCs; Adipose tissue-derived mesenchymal stem cells.

Trilineage differentiation was performed for the osteogenic, chondrogenic and adipogenic lineages in all samples. The negative control (non-induced) cells for each type of differentiation were negative for Alizarian Red, Oil Red O and Alician blue staining respectively (Fig 4 A, C, E).

In osteogenic differentiation, extracellular calcium deposition was clearly detected by Alizarin Red stain (Fig 4B) in AT-MSCs. Adipogenic differentiation was confirmed using Oil Red O that stained intracellular neutral lipid droplets (Fig 4D). Chondrogenic differentiation of AT-MSCs was also confirmed using Alician blue staining to detect proteoglycans in the extracellular matrix (Fig 4F).

### Isolated MSCs expressed CD29, CD44 and CD90, but not CD34 and MHC-II

**Fig 5:**
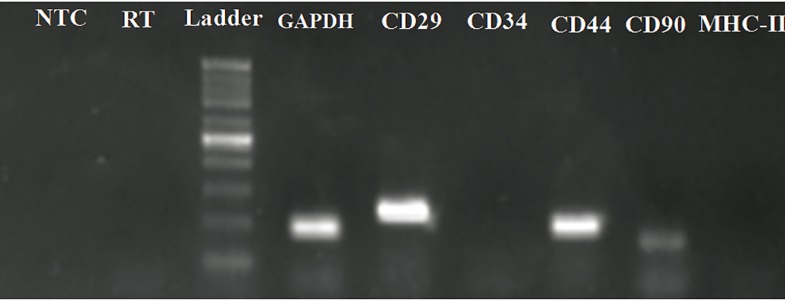
Expression of GAPDH, CD29, CD34, MHC-II, CD44 and CD90 mRNA of AT-MSCs was analyzed by RT-PCR. Representative ethidium bromide-stained gel electrophoresis revealed expression of GAPDH, CD29, CD44 and CD90. No expression of CD34 and MHC-II was observed. RT has mRNA instead of cDNA template in PCR to exclude DNA contamination and NTC is negative control. The sizes of the generated products were estimated by comparison with the mobility of the 100 bp DNA step ladder. GAPDH; Glyceraldehyde 3-phosphate dehydrogenase, CD; Cluster of differentiation, MHC; Major histocompatibility complex, AT-MSCs; Adipose tissue-derived MSCs; RT-PCR; Reverse transcriptase- polymerase chain reaction and NTC; Non-template control.

The mRNA for equine GAPDH acted as an internal positive control. So, we first assessed its amplification in all samples. Detection of a 183-bp fragment in all samples showed good RNA purification as well as suitable RT-PCR conditions. RTPCR results revealed expression of CD29, CD44 and CD90, and lack of expression of CD34 (hematopoietic marker) and MHC-II in all AT-MSCs (Fig 5).

## Discussion

Tendon and ligament injuries are a common cause of wastage among competitive horses and associated with failure to return to the previous level of performance along with an increased risk of re-injury. Cell-based therapies using MSCs have been reported in equine medicine with an increasing frequency in an attempt to improve the limited intrinsic capacity for complete self-repair of both cartilage and tendon after injury (3, 17). This study succeeds to isolate and identify equine MSCs from a little amount of adipose tissue using standard protocols. Based on requirements described for equine MSC characterization (14), firstly the isolated AT-MSCs were fibroblast-like and plastic-adherent, secondly they showed the capacity of tri-lineage differentiation *in vitro* and thirdly they expressed CD29, CD44 and CD90 but not CD34 and MHC-II.

The technique of adipose tissue collection as previously described (2, 11, 12) was performed from the base of the tail without any adverse effect. In previous studies, about 30-50 g adipose tissue was collected whereas we removed only 3-5 g tissue which was enough for cell isolation. About 80 to 300 million cells were obtained for each horse sample at the end of P3 which is a sufficient quantity for stem cell therapy (1, 3). Recently, it has been shown that half moon incision allows a more effective dissection of the subcutaneous tissue to collect the adipose tissue (18).

In the seventies, MSCs were isolated for the first time (19). MSCs are typically identified as plastic- adherent, spindle-shaped cells that grow in a monolayer and show a varying cellular morphology (20). Here, colonies of adherent fibroblastlike cells were observed in all cultures about day 7 after seeding. Morphology of isolated cells was similar to those reported by others (8, 21). In all 3 samples, a number of nodule-like cell aggregates were observed at P0. It has been shown that cells with similar phenotypes isolated from adipose tissue have some differences (22) and these aggregations may originate from a special population of cells in culture which should be more characterized. Based on growth curve results, the lag phase was short and cells rapidly started to enter the logarithmic phase. Although the growth rate of cells was reduced gradually after day 6, the cells did not reach the plateau phase within 8 days. This indicates high reproducibility of AT-MSCs in agreement with Burk et al. (8).

Doubling time (DT) of cells at passage 3 of all horses was between 40 to 46 hours. This is almost 2 days and much faster than the time for the duplication of human AT- MSCs reported at about 4 days (23). Schwarz et al. (24) reported the DT about 54 to 70 hours in horse, 54 to 65 hours in pigs and 48 to 68 hours in dogs. It suggests that differences in the duration of cell duplication among different species of mammals and even individuals of the same species is possible which can be due to heterogeneity of the starting population (22, 25) and/ or different composition of culture medium and growing conditions (24).

Tri-lineage differentiation is one of the minimal criteria for identification of MSCs (14, 15). Differentiation is a process which explicitly changes the cell in size, shape, membrane potential and metabolic activity caused by modifications in gene expression (14). According to the staining analysis, MSCs from the three horses differentiated into osteogenic, adipogenic and chodrogenic lineages similar to other studies (12, 26, 27).

Radcliffe et al. (28) studied the temporal expression changes of several genes (CD29, CD44, CD90, CD11a/CD18 and CD45RB) during establishment of equine MSC cultures, both at the mRNA and protein level. They found that at all culture time points tested, mRNA expression followed the same pattern as the cellular protein expression, indicating that gene expression analysis at the mRNA level can still be of great value. Our RT-PCR results revealed the expression of CD29 (â1 integrin), CD44 (Hyaluronate receptor) and CD90 (Thy-1) in equine AT-MSCs in agreement with other studies (13, 27, 29, 30). CD34, a surface marker of hematopoietic stem cells, was not detectable in isolated cells which shows that equine AT-MSC were not derived from circulating stem cells (31). However, the expression of CD34 in MSC is controversial. There are some conflicting results regarding its expression (28, 29). Moreover, our isolated cells did not express the MHCII antigen. Lack of expression of MHC-II, similar to other studies (7, 30), confirms that the isolated cells are less immunogenic and can be considered for allogenic grafts.

## Conclusion

The phenotypic characteristics of isolated ATMSCs in association with their multi-lineage differentiation potential and gene expression profile confirmed that the isolated cells are actually MSCs. This cell type can be considered as an appropriate candidate for cell therapies in equine regenerative medicine. In addition, we demonstrate that little amount of adipose tissue (3-5 g) is enough for MSC isolation. Nonetheless, further studies are warranted to identify different populations of adipose tissue multipotent stem cells and to clarify biological mechanisms involved in the active proliferation and cellular plasticity of equine MSCs before their extensive clinical use.
